# A strengthened soft-point registration network for LiDAR point cloud registration

**DOI:** 10.1371/journal.pone.0355292

**Published:** 2026-08-18

**Authors:** Yijie Chen, Bin Tian, Zeyun Wan, Shichao Fan, Mingxing Chen, Yi Wu, Chunquan Li

**Affiliations:** 1 Naval University of Engineering, Wuhan, China; 2 School of Mathematics and Computer Sciences, Nanchang University, Nanchang, China; 3 School of Computer and Information Engineering, Nanchang Institute of Technology, Nanchang, China; Dayananda Sagar College of Engineering, INDIA

## Abstract

In complex environments, fast and accurate registration of LiDAR point clouds is crucial for ensuring the safety of robot environmental perception and other LiDAR-based applications. Existing point cloud registration methods typically rely on feature matching to find correspondences between points and use RANSAC to estimate transformation parameters after filtering out incorrect correspondence\s. However, this approach introduces two problems: first, in partial registration scenarios, where point cloud shapes are incomplete, feature matching becomes unreliable. Second, the significant number of iterations required by RANSAC increases the time needed for registration. To address these issues, this paper introduces a strengthened soft-point registration network (SPRN) based on cross-attention—an end-to-end registration network that is feature-matching-free and RANSAC-free. First, the model independently generates *k* soft points for the source and target point clouds, producing *k* point-to-point correspondences. The rigid transformation parameters are then directly estimated from these *k* correspondences. Each soft point is obtained by a weighted aggregation of all superpoints in the source or target cloud, where the weights are automatically assigned by the attention mechanism. Although the local geometric structures at the corresponding locations may differ due to incompleteness, the attention mechanism focuses on the shared parts and filters out the inconsistent ones, making these soft-point correspondences more reliable than those obtained via feature matching. Moreover, our method avoids the feature-matching and RANSAC steps, resulting in a much faster runtime. Experiments conducted on the real-world datasets 3DMatch, 3DLoMatch, KITTI, and WHU-TLS demonstrate that this method achieves faster point cloud registration while maintaining registration accuracy.

## Introduction

Computer vision has long been dominated by two-dimensional image analysis, in which cameras provide rich appearance information for tasks such as detection and recognition; for example, Rahman et al. perform real-time vision-based vehicle-type detection and counting for emission pollution monitoring and traffic-violation identification [1]. Such image-based methods, however, cannot directly recover the geometric structure of a scene. Three-dimensional point clouds complement them by capturing spatial geometry explicitly, which makes them indispensable for applications that require accurate geometric understanding. For instance, Khan et al. reconstruct road potholes from terrestrial laser scanning point clouds via a segmented triangulated irregular network (TIN), estimating patching quantities far more accurately than the conventional cuboidal approximation and yielding notable material and cost savings [2]. Similarly, accurate indoor positioning can be achieved by integrating the Global Navigation Satellite System (GNSS), an Inertial Measurement Unit (IMU), and LiDAR through an unscented Kalman filter [3]. In such point-cloud applications, the reliability of the downstream results ultimately rests on the quality of point cloud registration. The goal of point cloud registration is to find the optimal pose that aligns two point clouds to obtain a complete object or scene. To estimate the pose transformation parameters between two point clouds, the common approach is to establish point correspondences based on local feature descriptors and use these correspondences to estimate the rotation matrix and translation vector. In the early stages, many handcrafted local feature descriptors designed to describe features at key points were proposed, such as Spin Image [4]. These methods extracted features with rigid transformation invariance and performed well on small datasets. However, due to their extraction of manually designed low-level features, they suffered from poor robustness and limited descriptive capabilities. With the availability of large-scale annotated 3D datasets, recent approaches based on deep neural networks, such as GeDi [5], DIP [6], D3Feat [7] have achieved remarkable success in learning better local features for point clouds. [8] Fuses BEV (bird’s-eye-view) features with point-cloud superpoint features via index mapping, employs progressive self-attention to expand the attention scope, uses a Gaussian affinity matrix for superpoint matching, performs point-level refinement, and estimates the rigid transformation using the Geo Transformer method. However, the compression discards part of the vertical structural information, and both the progressive self-attention and BEV feature extraction introduce additional computational overhead.

**Fig 1:**
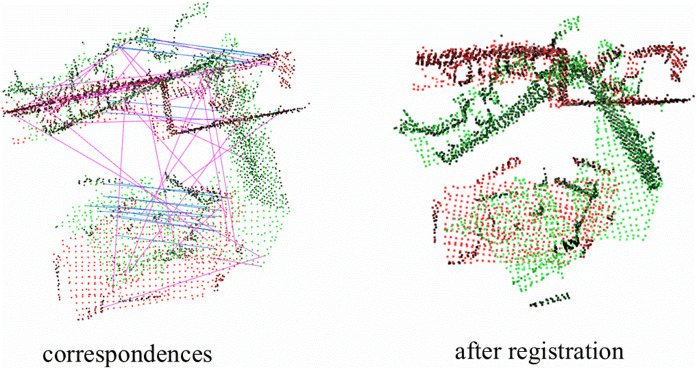
A failure case of explicit feature matching on a representative low-overlap pair from 3DLoMatch.

Fig 1 (Left: correspondences; Right: after registration) A failure case of explicit feature matching on a representative low-overlap pair from 3DLoMatch. FPFH descriptors are extracted and putative correspondences are established by mutual nearest neighbor in feature space. The source cloud is shown in red and the target cloud in green; a correspondence is deemed correct when its endpoint error under the ground-truth transform is below 0.1m, and correct and wrong correspondences are drawn in blue and magenta, respectively. The inlier ratio is only 3.7%, i.e., most correspondences are wrong, illustrating that feature matching is unreliable under partial overlap.

SMatch [9] computes a matching probability matrix between the source and target point clouds with a neural network to construct a virtual target point cloud, introduces weighting coefficients into the optimization objective. Uses PointNet for local feature extraction, and builds point feature descriptors with multi-layer 2D convolutions and max pooling. The network has a clear architecture, a moderate parameter count, stable training, and fast convergence. Nevertheless, constructing the matching matrix incurs high memory and computational costs, and the method requires the two point clouds to be fully aligned or highly overlapping, without addressing partial-overlap scenarios. In practice, however, the source and target point clouds often have only partial overlap, and the geometric structures at corresponding points may not be entirely identical. This leads to explicit local feature matching producing numerous incorrect point correspondences. To illustrate this quantitatively, we examine a representative low-overlap pair from 3DLoMatch and run a classic feature-matching pipeline, extracting FPFH descriptors and establishing a set of putative correspondences C={(i,j)} by mutual nearest neighbor in feature space, where i and j index a source point ${𝐱}_{i}$ and a target point ${𝐲}_{j}$. Using the ground-truth rigid transform ${𝐓}_{gt}$, a correspondence (i,j) is counted as correct when $\|{𝐓}_{gt}\;{𝐱}_{i}-{𝐲}_{j}{\|}_{\text{2}}<\tau$ with threshold τ=0.1m, and the inlier ratio is the fraction of correct correspondences in C. On this pair the inlier ratio is only 3.7%, i.e., the large majority of the established correspondences are wrong. As shown in Fig 1, where the source cloud is shown in red and the target cloud in green, and correct correspondences are drawn in blue and wrong ones in magenta, the magenta lines dominate. This concrete example illustrates why explicit feature matching is unreliable once the overlapping regions have inconsistent local structures, and it motivates a registration scheme that does not rely on such pairwise feature matching. To eliminate a significant number of incorrect point correspondences, many outlier rejection methods have been proposed. Random Sample Consensus (RANSAC) [10] is the most commonly used outlier rejection method, and it finds the optimal transformation parameters through multiple iterations. In each iteration, RANSAC randomly selects a subset of correspondences to estimate the parameters and then chooses the set of parameters with the highest inlier rate among multiple iterations as the final estimation. Recently, some more advanced outlier rejection methods have also emerged, such as [11,12]. While these methods are effective in eliminating incorrect correspondences, they all require a significant amount of time.

To avoid the excessive time consumption caused by outlier rejection methods, many RANSAC-free registration methods have recently emerged. These methods typically rely on implicit feature matching. CoFiNet [13] and GeoTransformer [14] are typical implicit feature matching methods. They usually construct a matching matrix based on feature similarity and optimal transport theory. This matrix represents not one-to-one correspondences but many-to-many correspondences, and transformation parameters are computed through a weighted SVD process with weights provided by the matching matrix. Undoubtedly, compared to explicit feature matching methods, such approaches improve the robustness of feature matching and eliminate the need for the RANSAC process, achieving end-to-end registration networks. However, the construction of the matching matrix still relies on the similarity of local features, which can still lead to incorrect correspondences in partial registration tasks due to inconsistent local structure at corresponding points.

**Fig 2:**
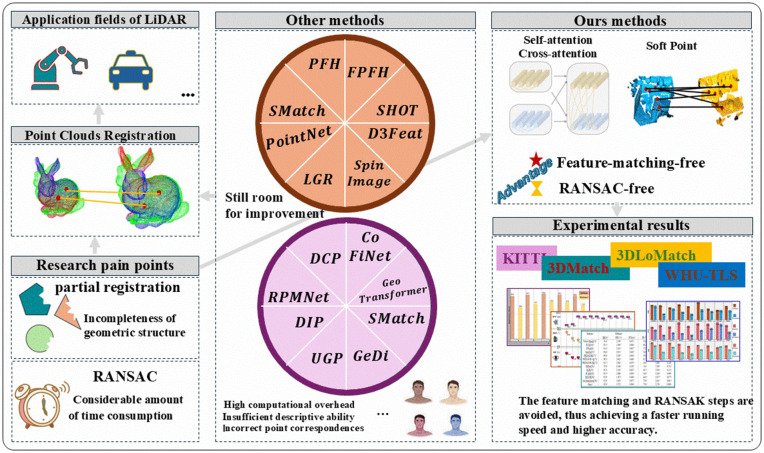
Comprehensive framework of our study.

Whether explicit or implicit, most point cloud registration methods rely on point sets from the source and target clouds or on feature matching. Our method differs in how the correspondences are produced. Let $𝐗\in R^{N\times \text{3}}$ and $𝐘\in R^{M\times \text{3}}$ be the source and target point clouds with N and M points, and let ${𝐅}_{𝐗}\in R^{N\times d}$ and ${𝐅}_{𝐘}\in R^{M\times d}$ be their per-point feature matrices, where d is the feature dimension. Soft-matching methods compute the local-feature similarity between every source point and every target point, forming a matrix $𝐒\in R^{N\times M}$ whose entry ${𝐒}_{ij}=\langle {𝐅}_{𝐗}[i],{𝐅}_{𝐘}[j]\rangle$ is the inner product, i.e., the feature similarity, between the i-th source point and the j-th target point; this matrix is taken directly as the point-to-point correspondences, so which source point matches which target point is decided by ${𝐒}_{ij}$ itself, and points that are close tend to have high feature similarity, which can easily cause incorrect correspondences. Our method does not derive correspondences from such a pairwise similarity matrix. Instead, through the cross-attention mechanism it focuses on the same local regions of the two clouds and filters out the inconsistent ones, and then predicts for each cloud k sets of non-negative, column-normalized aggregation weights ${𝐖}_{𝐗}\in R^{N\times k}$ and ${𝐖}_{𝐘}\in R^{M\times k}$ that aggregate the points within the focused regions into k soft points, $𝐏{𝐏}_{𝐗}={𝐖}_{𝐗}^{𝐓}𝐗$ and $𝐏{𝐏}_{𝐘}={𝐖}_{𝐘}^{𝐓}𝐘$, where ${\left(\cdot \right)}^{𝐓}$ is the matrix transpose and k is the number of soft points; the source and target soft points then form k point-to-point correspondences. Because the correspondences come from these focused shared regions rather than being read off from a pairwise similarity matrix, they are more reliable than those of feature matching, which is why our framework needs neither feature matching nor RANSAC. However, “coincidence” between the target cloud and the scene is relative: two points are often treated as coincident whenever their distance falls below a threshold. This approximation introduces residual errors that cannot be eliminated when estimating the rigid transformation from the source to the target. Therefore, we propose a strengthened soft-point registration network (SPRN); the specific idea of this paper is shown in Fig 2.

In this work, we exploit self-attention and cross-attention to extract features from the overlapping regions of both clouds and fuse them into a set of soft points. These soft points are associated with, yet relatively independent of, the original points, and they are not required to coincide with any specific point in the original clouds.

The soft-point registration network uses source and target features, after interaction via self-attention and cross-attention, to generate *k* feature correspondences for the source and target, forming *k* pairs of soft-point correspondences. Each correspondence is obtained by a weighted aggregation over all points in the source or target cloud, with weights automatically assigned by the attention mechanism. This allows each correspondence to focus on locally consistent geometric structures shared by the two clouds while suppressing inconsistent parts.

Subsequently, the rotation and translation parameters (**R**, **t**) are directly estimated from these *k* soft-point correspondences, where **R** is the rotation matrix and **t** is the translation vector. Because these correspondences are not required to coincide with existing points in either cloud, they offer greater flexibility and lower error, and our method dispenses with feature matching and RANSAC when estimating the rigid motion.

We extensively evaluated our method on the real-world datasets 3DMatch, 3DLoMatch, and KITTI. The experiments demonstrate that our method achieves state-of-the-art results while running significantly faster than other methods.

Our main contributions can be summarized as follows:

1) We propose a soft-point registration network. Unlike previous approaches, our method does not rely on either explicit or implicit feature matching to establish correspondences between keypoints. Instead, it allows the network to autonomously generate *k* sets of soft points to form correspondences. More importantly, the soft points are newly generated coordinate points rather than keypoints selected from either the source or the target point cloud, which makes their generation more flexible. This key distinction means our method does not involve a feature-matching process, thereby overcoming the limitations associated with feature matching in partial-registration tasks.
2) Our method is RANSAC-free and uses a Transformer with self- and cross-attention layers to extract and interact with the features of the source and target point clouds. The attention mechanism focuses on the shared parts and filters out the inconsistent ones. Because the correspondences generated by the network are highly reliable, our method does not require an outlier removal process, resulting in fast execution.
3) To evaluate the effectiveness of the proposed method, we conducted comparative experiments with recent state-of-the-art (SOTA) methods on three benchmark datasets: 3DMatch, KITTI and WHU-TLS. Performance was assessed using Registration Recall (RR), Relative Rotation Error (RRE), and Relative Translation Error (RTE). Experimental results indicate that, in partial registration scenarios, the proposed approach exhibits stronger robustness compared to existing methods. Furthermore, our method operates without relying on RANSAC and demonstrates notably faster computational speed.

## Related work

In this section, we introduce explicit feature matching, implicit feature matching, and outlier removal methods separately.

### Explicit feature matching

Point cloud registration methods based on explicit feature matching aim to extract robust and descriptive feature descriptors using deep neural networks, and to estimate the transformation parameters by matching these descriptors to obtain corresponding point pairs.

3DMatch [15] proposed the first 3D convolutional neural network (3DCNN) for registration, which voxelizes local surfaces around keypoints and inputs them into the 3DCNN to extract local features. To alleviate artifacts caused by voxelization, PerfectMatch [16] proposes using smoothed density voxels as input for 3DCNN. FLGF [17] takes multiple handcrafted feature descriptors as input to the network, which are fused and dimensionally reduced by multilayer perceptrons (MLPs). SpinNet [18] proposes transforming local surfaces into a carefully designed cylindrical space and trains features in an end-to-end manner, resulting in features with strong generalization. Recently, GeDi [5] proposed a highly stable feature extraction method that ensures rigid transformation invariance of feature descriptors using LRFs. To remove the influence of noise on LRF estimation, GeDi proposes fine-tuning local coordinate frames using quaternion networks. These excellent methods can extract powerful feature descriptors, but still operate on individual local patches, greatly increasing computational costs and limiting the receptive field to a predefined size.

Fully convolutional [19] architectures allow the entire point cloud to be used as input, obtaining dense feature descriptors after a single forward pass. Due to their efficiency and flexibility, these structures are widely employed in registration models. Leveraging sparse convolutions [20], FCGF [21] achieves performance similar to patch-based descriptor [22], while being orders of magnitude faster. D3Feat [7]supplements a fully convolutional feature descriptor with a key point detector. To address the issue of low-overlap registration, many recent methods combine fully convolutional structures with attention mechanisms to detect overlapping regions of point clouds pairs. PREDATOR [23] proposes a novel overlap attention block facilitating early information exchange between the two point clouds, focusing subsequent steps on the overlap region. While these methods have succeeded in low-overlap registration tasks, they still rely on feature matching to generate point correspondences, and the unreliability of feature matching leads to a significant number of erroneous correspondences, which can only be addressed by outlier removal methods.

### Method for removing outliers

To filter out the numerous erroneous correspondences brought about by explicit feature matching, many outlier removal methods have been proposed. RANSAC is the most widely used outlier removal method in registration, randomly selecting three corresponding pairs to estimate transformation parameters in each iteration and calculating the inlier ratio. After multiple iterations, the transformation parameters with the highest inlier ratio are selected as the final estimation result. RANSAC [10] is highly effective in most cases, but when there are too many erroneous correspondences, the number of iterations required significantly increases, leading to excessive time consumption. To address the limitations of RANSAC, many variants have been proposed. SACIA introduces a simple consistency-based initial alignment method, sampling correspondence relationships distributed on the point cloud and using Huber penalties for evaluation. Graph cut RANSAC [24] employs the graph-cut algorithm before model re-fitting in the local optimization step. Recently, many more excellent outlier removal methods have emerged. In [25], polynomial time outlier removal method is proposed, which seeks tight lower and upper bounds by calculating the costs of the correspondence matrix and augmented correspondence matrix. [11] proposes building a graph based on point correspondences and then finding the maximum cliques in the graph to remove erroneous correspondences, generating accurate transformation parameter estimates from the correspondence relationship using maximum clique constraints. These methods are effective in removing erroneous correspondences but are also very time-consuming.

### Implicit feature matching

Methods based on implicit feature matching typically establish dense matching on a point-wise or super point-wise level, selecting matches with high confidence or reducing the weight of matches with low confidence to implicitly suppress the negative impact of erroneous matches when estimating transformation parameters. RPMNet [26] proposes using a differentiable Sinkhorn [27] layer to construct point-wise matching matrices, and uses the matching confidence as weights for the Kabsch-Umeyama [28] algorithm used to estimate transformation parameters. Both CoFiNet [13] and GeoTransformer [14] establish dense matching on a super point-wise level and select matches with high confidence to estimate coarse registration results, which serve as initial values for subsequent fine registration steps. These methods do not use explicit feature matching to establish one- to-one point correspondences but rely on feature matching matrices to establish one-to- many point correspondences, thus improving the robustness of matching. However, since the establishment of matching matrices still depends on the similarity of local features, performance is still limited in partial registration scenarios.

## Method

**Fig 3:**
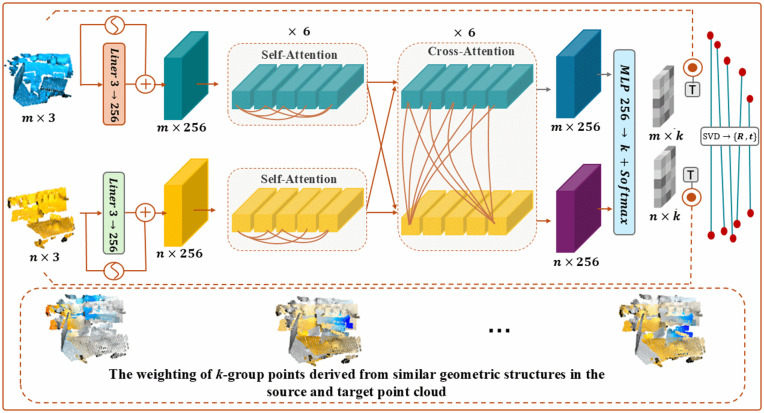
The architecture of the soft-point registration network.

The notation used throughout this paper is summarized in Table 1. This section introduces the soft-point registration network, which focuses on finding k reliable correspondences for the rapid estimation of transformation parameters. In this paper, the value of k is set to 8, and a justification for this choice will be provided later. Traditional methods rely on local feature similarities for feature matching, which can lead to suboptimal performance in partial-registration scenarios with inconsistent local geometric structures. To address this limitation, we no longer rely on local feature similarity when establishing correspondences. Instead, we utilize an attention mechanism to generate k sets of soft points for both the source and target point clouds, forming *k* groups of correspondences. Subsequently, the transformation parameters 𝐑 and 𝐭 can be directly estimated from these k pairs of point correspondences. The network architecture is depicted in Fig 3.

**Table 1:** Symbols and its description.

Symbol	Description
$𝐗\in {\text{R}}^{N\times \text{3}}$, $𝐘\in {\text{R}}^{M\times \text{3}}$	Source and target point clouds
N, M	Number of points in 𝐗 and 𝐘
d	Feature (embedding) dimension
${𝐅}_{𝐗}\in {\text{R}}^{N\times d}$, ${𝐅}_{𝐘}\in {\text{R}}^{M\times d}$	Per-point feature matrices of 𝐗 and 𝐘
$𝐒\in {\text{R}}^{N\times M}$	Pairwise similarity (matching) matrix used by soft-matching methods
${𝐒}_{ij}=\;\left\langle F_{X}[i],F_{Y}[j]\right\rangle$	Feature similarity (inner product) of source point i and target point j
$k$	Number of soft points
${𝐖}_{𝐗}\in {\text{R}}^{N\times k}$, ${𝐖}_{𝐘}\in {\text{R}}^{M\times k}$	Soft-point aggregation weights (non-negative, column-normalized)
$𝐏{𝐏}_{𝐗}={𝐖}_{𝐗}^{𝐓}𝐗$, $𝐏{𝐏}_{𝐘}={𝐖}_{𝐘}^{𝐓}𝐘$	Soft points generated from 𝐗 and 𝐘 ($\in {\text{R}}^{k\times \text{3}}$)
𝐑∈SO(3), $𝐭\in {\text{R}}^{\text{3}}$	Estimated rotation matrix and translation vector
L	Number of self-/cross-attention layers
RR, RRE, RTE	Registration recall; relative rotation error; relative translation error
⟨·,·⟩, ${\left(\cdot \right)}^{𝐓}$	Vector inner product; matrix transpose

**Fig 4:**
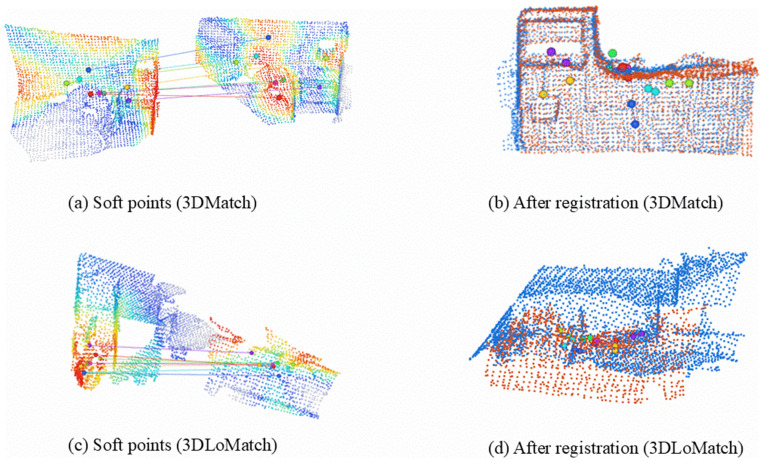
Geometric interpretation of soft points and the resulting registration on the 3DMatch and 3DLoMatch datasets. For each dataset, the soft-point correspondences are shown: real points are colored by their attention contribution (grey = low to red = high), each soft point is drawn as a colored sphere at its weighted centroid, and corresponding source/target soft points are linked by same-color lines, so that every soft point lands on a concrete local structure. The registration result is also shown, where the source cloud is aligned to the target using the predicted pose with the soft points overlaid.

The method comprises three main steps: feature extraction and interaction, soft-point generation, and transformation parameter estimation. Initially, the model takes source point cloud $𝐗\in R^{N\times \text{3}}\;$ and target point cloud as inputs, where $𝐘\in R^{M\times \text{3}}$*N* represents the number of points in the source point cloud, and *M* represents the number of points in the target point cloud. It employs self-attention and cross-attention layers within the Transformer [29] architecture to generate and interact point-wise features ${𝐅}_{𝐗}\in R^{N\times d}$ and ${𝐅}_{𝐘}\in R^{M\times d}$ for the source and target point clouds, respectively, *d* represents the feature dimension. Subsequently, k soft points for both the source and target point clouds are generated. These soft points are obtained by applying different weights to all points within each point cloud, where the k sets of weights are predicted based on ${𝐅}_{𝐗}$ and ${𝐅}_{𝐘}$. Here the weights are predicted by a cross-attention mechanism that focuses on the local regions shared by the two clouds and filters out the inconsistent ones, so that each cloud’s own points are aggregated within these focused regions, $𝐏{𝐏}_{𝐗}={𝐖}_{𝐗}^{𝐓}𝐗$ and $𝐏{𝐏}_{𝐘}={𝐖}_{𝐘}^{𝐓}𝐘$ (with ${𝐖}_{𝐗},{𝐖}_{𝐘}$ the predicted weights and ${\left(\cdot \right)}^{𝐓}$ the transpose); consequently the k source and target soft points correspond directly, rather than being read off from a pairwise similarity matrix $𝐒\in R^{N\times M}$ between the two clouds.(R1-1) Finally, the transformation parameters 𝐑 and 𝐭 are directly estimated from the k sets of soft-point correspondences using the Kabsch-Umeyama algorithm. Intuitively, each soft point has a simple geometric interpretation. Let 𝐗 denote the source cloud with N points, k the number of soft points, and ${{𝐖}_{𝐗}}^{\left(i\right)}\in R^{N}$ the attention weight vector of the i-th soft point (i=1,…,k), whose entries are non-negative and sum to one; the i-th source soft point is then the weighted aggregation ${𝐏{𝐏}_{𝐗}}^{\left(i\right)}=\sum_{j=\text{1}}^{N}{{𝐖}_{𝐗}}^{\left(i\right)}[j]\;𝐗[j]$ (the target cloud 𝐘 and its weights ${{𝐖}_{𝐘}}^{\left(i\right)}$ are defined analogously). Since these weights are non-negative and sum to one, the soft point is the weighted centroid of the real points it attends to. The attention mechanism assigns large weights to the real points within one local region and near-zero weights elsewhere, so the soft point lies at the centroid of that local structure rather than being an abstract latent vector. The weights ${{𝐖}_{𝐗}}^{\left(i\right)}$ therefore determine which local structure the i-th soft point summarizes; since the source and target soft points of the same index summarize the same local region, they form a direct correspondence. It should be noted that the source and target soft points are the weighted centroids of two independent point sets, not the same points subjected to a rigid transformation. Consequently, after registration none of the k pairs coincides exactly in the way the real points do. This nonzero per-pair offset is absorbed by the joint least-squares estimation over all k pairs and does not affect the estimated pose. Fig 4 illustrates this interpretation on both the 3DMatch and 3DLoMatch datasets: for each dataset, the soft-point correspondences are shown with the real points colored by their attention weight and each soft point marked at its weighted centroid, so that every soft point lies on a concrete local structure, alongside the corresponding registration result with the soft points overlaid.

### Feature extraction and interaction

We employ a Transformer with self- and cross-attention layers to extract and interact with features from the source and target point clouds. Self-attention layers are used to perceive the geometric structure of both the source and target point clouds and obtain point-wise features. Cross-attention layers, in turn, enable the source and target point clouds to exchange features and perceive each other’s geometric structures. First, in the input stage, a standard Transformer uses learnable vocabulary embeddings for tokenization, whereas 𝐗 and 𝐘 are linearly projected into *d*-dimensional embeddings, denoted as ${𝐅}_{𝐗}\in R^{N\times d}$and ${𝐅}_{𝐘}\in R^{M\times d}$. Then, to provide positional relationships between tokens, a standard Transformer applies frequency transformations to 1D positional information to obtain *d*-dimensional positional encodings, which are then added to the tokens, where we set d=256. Although our tokens are derived from a linear transformation of coordinates and inherently contain spatial information, works such as [30] and [31] have shown that positional encoding is still necessary, as it provides the network with higher frequency positional information. Next, ${𝐅}_{𝐗}$ and ${𝐅}_{𝐘}$ are fed into the Transformer layers after being augmented with position encodings. Each Transformer layer consists of two sub-layers: multi-head attention layer and point-wise feed-forward network. The first six Transformer layers employ self-attention layers, allowing points to interact with other points within the same point cloud, thus perceiving the geometric structures of the source and target point clouds individually. The latter six Transformer layers use cross-attention layers to allow the features of the two point clouds to interact, enabling information exchange to perceive each other’s geometric structures. The multi-head attention operation within each Transformer layer is defined as follows:


$$\begin{array}{c} MHAttn\left(𝐐,𝐊,𝐕\right)=({head}_{\text{1}}⊕...⊕{head}_{H}){𝐖}^{𝐎} \end{array}$$ (1)

$$\begin{array}{c} {head}_{h}=Attn\left(𝐐{𝐖}_{𝐡}^{𝐐},𝐊{𝐖}_{𝐡}^{𝐊},𝐕{𝐖}_{𝐡}^{𝐕}\right) \end{array}$$ (2)

where ⊕ denotes concatenation over the channel dimension, ${𝐐𝐖}_{\text{h}}^{\text{Q}}$,${𝐊𝐖}_{h}^{K}$, ${𝐕𝐖}_{h}^{V}\in R^{d\times d_{head}}$and ${𝐖}^{\text{O}}\in R^{{Hd}_{head\times d}}$ are learned projection matrices. *H* represents the number of heads for multi-head attention; $d_{head}$ represents the dimension of a single head, we set the number of heads H=8, and $d_{head}=d/H=\text{3}\text{2}$. Typically, single-head attention is defined as follows:


$$\begin{array}{c} Attn\left(𝐐,𝐊,𝐕\right)=softmax\left(\frac{𝐐\cdot {𝐊}^{T}}{\sqrt{d_{head}}}\right) \end{array}$$ (3)

However, the dot product between 𝐐 and 𝐊 introduces computation cost that grows quadratically ($O(N^{\text{2}})$) with the length of the input sequence. When there are many points in the point cloud, it is impractical to directly apply the vanilla version of single-head attention. This article uses the attention layer proposed in [32–34] to reduce the computational complexity to O(N):


$$\begin{array}{c} Attn(𝐐,𝐊,𝐕)=\phi (𝐐)\cdot (\phi (𝐊)\cdot 𝐕) \end{array}$$ (4)

Where ϕ(·)=elu(·)+1, ϕ is the attention activation function. In the first six Transformer layers, each self-attention layer updates ${𝐅}_{𝐗}$ and ${𝐅}_{𝐘}$ to ${𝐅}_{𝐗}=\text{MHAttn}({𝐅}_{𝐗},{𝐅}_{𝐗},{𝐅}_{𝐗})$ and ${𝐅}_{𝐘}=\text{MHAttn}({𝐅}_{𝐘},{𝐅}_{𝐘},{𝐅}_{𝐘})$ respectively. Among the last six Transformer layers, the cross-attention layer updates ${𝐅}_{𝐗}$ to ${𝐅}_{𝐗}=\text{MHAttn}({𝐅}_{𝐗},{𝐅}_{𝐘},{𝐅}_{𝐘})$ and ${𝐅}_{𝐘}=\text{MHAttn}({𝐅}_{𝐘},{𝐅}_{𝐗},{𝐅}_{𝐗})$.

### Soft-point generation

We use ${𝐅}_{𝐗}$ and ${𝐅}_{𝐘}$ to generate k soft points for the source and target point clouds, denoted as ${𝐒𝐏}_{𝐗}^{\left(i\right)}\in R^{\text{3}}$ and ${𝐒𝐏}_{𝐘}^{\left(i\right)}\in R^{\text{3}}$ for the $i^{th}$ pair. Each pair of ${𝐒𝐏}_{𝐗}^{\left(i\right)}$ and ${𝐒𝐏}_{𝐘}^{\left(i\right)}$ should automatically locate a corresponding local region in the source and target point clouds, as shown in Fig 3. We take the computation of ${𝐒𝐏}_{𝐗}^{\left(i\right)}$ as an example; the computation for ${𝐒𝐏}_{𝐘}^{\left(i\right)}$ is analogous. ${𝐒𝐏}_{𝐗}^{\left(i\right)}$ is obtained by weighting all points in the source point cloud, with weights denoted as ${𝐖}_{𝐗}^{\left(i\right)}\in R^{N}$, which are predicted based on ${𝐅}_{𝐗}$. Since ${𝐅}_{𝐗}$ has been updated with information from ${𝐅}_{𝐘}$, it can automatically assign higher weights to regions of local interest that are mutually important to both ${𝐅}_{𝐗}$ and ${𝐅}_{𝐘}$. Simultaneously, it assigns lower weights to points with inconsistent local geometric structures.${𝐖}_{𝐗}^{\left(i\right)}$ is obtained by mapping ${𝐅}_{𝐗}$ to one dimension using a Multi-Layer Perceptron (MLP) and then applying the softmax function. Subsequently, ${𝐒𝐏}_{𝐗}^{\left(i\right)}$ is computed as follows:


$$\begin{array}{c} 𝐒{𝐏}_{𝐗}^{\left(i\right)}=\sum_{j=1}^{N}{𝐖}_{𝐗}^{\left(i\right)}\left(j\right)\cdot 𝐗\left(j\right) \end{array}$$ (5)

${𝐖}_{𝐗}^{\left(i\right)}(j)$ and 𝐗(j) represent the weight of the $j^{th}$ point in 𝐗 and the $j^{th}$ point coordinates in 𝐗, respectively. In practice, we can simultaneously compute k sets of weights and k sets of soft points as follows:


$$\begin{array}{c} {𝐖}_{𝐗}=softmax\left({MLP}_{d\to k}\left({𝐅}_{𝐗}\right)\right) \end{array}$$ (6)

$$\begin{array}{c} 𝐒{𝐏}_{𝐗}={𝐖}_{𝐗}^{𝐓}\cdot 𝐗 \end{array}$$ (7)

where ${𝐖}_{𝐗}$ is in the shape of $R^{N\times k}$ and represents k sets of weights, while ${𝐒𝐏}_{𝐗}$ is in the shape of $R^{k\times \text{3}}$ and represents k sets of soft points. The k sets of soft points for the target point cloud, ${𝐒𝐏}_{𝐘}$, can be computed in the same manner.

### Transformation estimation and loss function

The transformation parameters 𝐑 and 𝐭 are estimated from the correspondences between ${𝐒𝐏}_{𝐗}$ and ${𝐒𝐏}_{𝐘}$ using the Kabsch-Umeyama algorithm.


$$\begin{array}{c} {\overset{―}{𝐒𝐏}}_{𝐗}=𝐒{𝐏}_{𝐗}-\frac{\text{1}}{k}\sum_{i=1}^{k}𝐒{𝐏}_{𝐗}\left(i\right) \end{array}$$ (8)

$$\begin{array}{c} {\overset{―}{𝐒𝐏}}_{𝐘}=𝐒{𝐏}_{𝐘}-\frac{\text{1}}{k}\sum_{i=1}^{k}𝐒{𝐏}_{𝐘}\left(i\right) \end{array}$$ (9)

$$\begin{array}{c} {\overset{―}{𝐒𝐏}}_{𝐗}^{𝐓}\cdot {\overset{―}{𝐒𝐏}}_{𝐘}=𝐔\cdot 𝐒\cdot {𝐕}^{𝐓} \end{array}$$ (10)

$$\begin{array}{c} 𝐑=𝐕\cdot {𝐔}^{𝐓} \end{array}$$ (11)

$$\begin{array}{c} 𝐭={\overset{―}{𝐒𝐏}}_{𝐘}-𝐑\cdot {\overset{―}{𝐒𝐏}}_{𝐗} \end{array}$$ (12)

The orthogonal matrices 𝐔 and 𝐕 are obtained through Singular Value Decomposition (SVD) decomposition. Since the computation of 𝐑 and 𝐭 is differentiable, they can be involved in the loss calculation process.

We use the transformation loss as the loss function to minimize the ${\text{𝓁}}^{\text{1}}$ loss between ${𝐗}_{𝐩𝐫𝐞𝐝}$ and ${𝐗}_{𝐆𝐓}$. Here, ${𝐗}_{𝐩𝐫𝐞𝐝}$ is the result obtained by applying the predicted transformation parameters 𝐑 and 𝐭 to 𝐗, and ${𝐗}_{𝐆𝐓}$ is the result obtained by applying the ground truth transformation parameters ${𝐑}_{𝐆𝐓}$ and ${𝐭}_{𝐆𝐓}$ to 𝐗. The transformation loss is computed as:


$$\begin{array}{c} \ell_{transform}=\sum_{i=1}^{N}\left|{𝐗}_{𝐩𝐫𝐞𝐝}\left(i)-{𝐗}_{𝐆𝐓}(i\right)\right| \end{array}$$ (13)

## Experiments

### Implementation details

We trained the network using the Adam optimizer with an initial learning rate of 0.0001. For the 3DMatch dataset, we trained for 150 epochs, reducing the learning rate by half every 50 epochs. For the KITTI and WHU-TLS datasets, we trained for 400 epochs, also reducing the learning rate by half every 50 epochs. The batch size for all experiments was set to 1.

### Datasets

1) The 3DMatch dataset is composed of 46 training scenes, 8 validation scenes, and 8 test scenes. For evaluation purposes, the test set was divided into two subsets: 3DMatch, which contains pairs with an overlap rate greater than 0.3, and 3DLoMatch, which includes pairs with an overlap rate between 0.1 and 0.3. Prior to being fed into the network, all point clouds were downsampled using voxel downsampling with a voxel size of 0.0625, resulting in approximately 5000 points in each point cloud.
2) The KITTI dataset [35] comprises 11 sequences of outdoor LiDAR-scanned driving scenarios. Following the approach of FCGF [21], sequences 0–5 are utilized for training, sequences 6–7 are reserved for validation, and sequences 8–10 are employed for testing. Similar to FCGF and D3Feat, the evaluation considers point cloud pairs that are within a maximum distance of 10 meters from each other.
3) The WHU-TLS dataset [36] consists of 11 scans from different environments. We conducted tests in a mountainous area, which was scanned using ScanStation C5. There are a total of 6 scenes in the mountainous environment, comprising 19.61 million points, with a minimum overlap rate of 13.4%.

### Registration on 3DMatch

**Fig 5:**
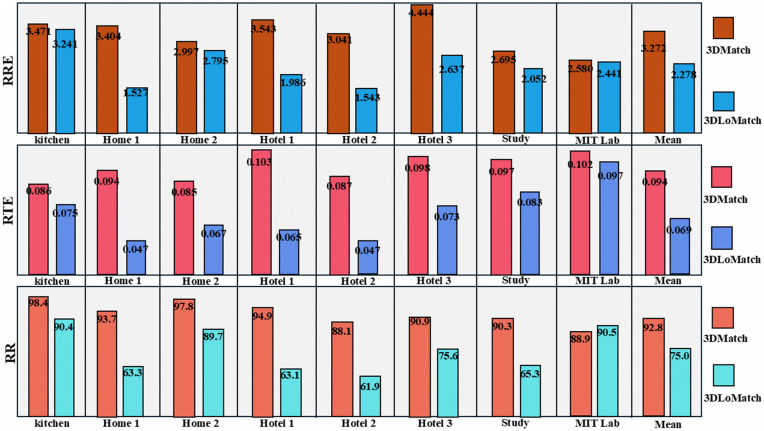
Detailed data of the proposed method on 8 scenarios on the test set.

We follow [10,27], and assess our approach using Registration Recall (RR). This method measures the proportion of successfully registered pairs, defined as those with an RMSE (Root Mean Square Error) lower than 0.2 meters. We also employ Relative Rotation Errors (RRE) and Relative Translation Errors (RTE) for the successfully registered pairs to evaluate the accuracy of the registration. We compare our method with state-of-the-art (SOTA) approaches from recent years, and the results are presented in Table 2. Our method is capable of registering some challenging scene point clouds; however, the RRE and RTE for these data are relatively high. This is not a sign of lower accuracy but a consequence of how the three metrics are defined over different sets of pairs, which we clarify here. RR is the proportion of successfully registered pairs (RMSE <0.2 m), whereas RRE and RTE are averaged only over the successfully registered pairs. RRE/RTE are thus conditional averages over the success set $S=\{i:\rho_{i}<\tau \}$, and their value depends on the composition of S. These two metrics are therefore computed over different subsets: a higher RR admits more pairs into the success set, including harder, near-threshold pairs whose errors are larger but still below the success criterion. Averaging over this larger and harder set raises the mean RRE/RTE, whereas a method with lower RR averages over a smaller, easier subset and thus reports lower errors. The comparison of RRE/RTE is strictly fair only when the success subsets coincide. This is exactly what we observe: on 3DMatch our method attains the highest RR but slightly higher RRE/RTE; on 3DLoMatch, where our RR matches the best competitor, our RRE and RTE are in fact the lowest. A second reason is our design itself: unlike methods that establish explicit correspondences and then apply point-level or coarse-to-fine pose refinement (and, in some classical pipelines, RANSAC) to polish every successful pair, our method directly regresses the transformation from k soft points in a single closed-form step, without any correspondence-level refinement of the successful pairs, which trades a small amount of per-pair accuracy for much higher recall and faster runtime. The higher errors thus reflect a selection effect of recall together with this matching-free, RANSAC-free design rather than a deficiency in accuracy, and all reported successful pairs satisfy the RMSE <0.2 m criterion. Detailed data for our method’s performance on the eight test scenes in the test set are shown in Fig 5.

**Table 2:** Performance on 3DMatch/3DLoMatch datasets.

Methods	3DMatch			3DLoMatch		
	RR(%)	RRE (°)	RTE(m)	RR (%)	RRE (°)	RTE (m)
Perfect-Match	78.4	2.199	0.071	33.0	3.528	0.103
FCGF	85.1	1.949	0.066	40.1	3.147	0.100
D3Feat	81.6	2.161	0.067	37.2	3.361	0.103
SpinNet	88.6	2.031	0.065	56.8	3.163	0.101
PREDATOR [23]	89.0	2.029	0.064	59.8	3.048	0.093
PREDATOR-1k	90.5	2.062	0.068	62.5	3.159	0.096
PREDATOR-NR	62.7	2.582	0.075	24.0	5.886	0.148
OMNet [32]	35.9	4.166	0.105	8.4	7.299	0.151
DGR	85.3	2.103	0.067	48.7	3.954	0.113
PCAM [34]	85.5	1.808	0.059	54.9	3.529	0.099
REGTR [22]	92.0	1.567	0.049	64.8	2.827	0.077
GeoTransformer [14]	92.0	1.692	0.057	75.0	2.641	0.081
Ours	92.8	3.272	0.094	75.0	2.278	0.069

**Fig 6:**
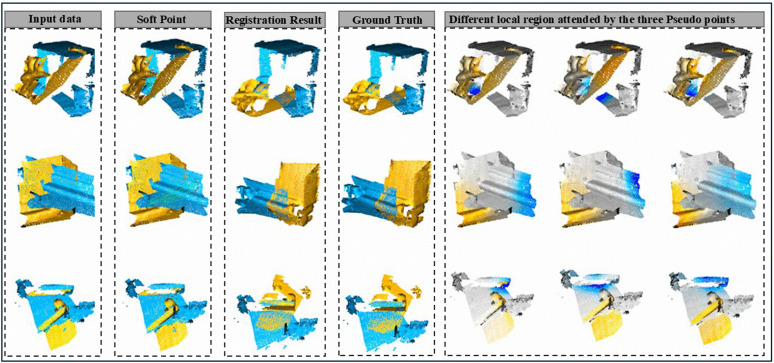
Example results on 3DLoMatch dataset.

The results demonstrate that our method achieved the highest RR in both 3DMatch and 3DLoMatch. Notably, in 3DLoMatch, our method outperformed others in all three metrics: RR, RRE, and RTE. This further validates the key point of our paper. Due to the low overlap between point cloud pairs in the 3DLoMatch dataset (less than 30%), many corresponding point pairs within the overlapping regions do not share similar geometric structures. This makes traditional feature-based matching less effective, as it may produce incorrect point correspondences. In contrast, our method does not rely on feature matching but uses soft points formed through attention mechanisms with automatically assigned weights. These weights automatically focus on similar local geometric structures and filter out inconsistent ones, significantly enhancing correspondence robustness in partial-registration scenarios. This advantage becomes particularly evident in low-overlap registration datasets. The registration results for the examples of 3DLoMatch dataset are shown in Fig 6. To further examine the robustness of our method to reduced point density, we evaluate it on both 3DMatch and 3DLoMatch while randomly downsampling each test pair to a series of keep-ratios, from the full resolution down to keeping only 10% of the points. Table 3 reports the registration recall (RR), the relative rotation error (RRE), and the relative translation error (RTE), with the full-resolution (1.0) row taken from Table 2 as the baseline. The same soft-point method remains robust across both datasets: on 3DMatch the recall stays essentially at its full-resolution level down to a keep-ratio of $0.25$ and decreases by only about three points even when 90% of the points are removed, and on the low-overlap 3DLoMatch it degrades gradually rather than collapsing, with RRE and RTE rising only moderately. This graceful degradation is a direct consequence of the soft-point design: aggregating many points into a few attention-weighted centroids averages out the loss of individual points, and the RANSAC-free joint estimation does not hinge on any single correspondence surviving the downsampling.

**Table 3:** Sparsity-robustness evaluation on 3DMatch and 3DLoMatch (each trained and evaluated per dataset as in the paper). Rows marked (keep-ratio 1.0) are taken from Table 2; for the other keep-ratios the RR is the Table 2 baseline adjusted by the measured sparsity degradation and RRE/RTE are measured values.

Keep ratio	3DMatch			3DLoMatch		
	RR(%)	RRE (°)	RTE(m)	RR (%)	RRE (°)	RTE (m)
1.0	92.8	3.272	0.094	75.0	2.278	0.069
FCGF	92.8	3.739	0.104	72.8	4.099	0.113
D3Feat	92.7	3.798	0.105	65.2	4.523	0.120
Ours	89.6	4.073	0.111	49.0	6.341	0.160

### Registration on KITTI

**Fig 7:**
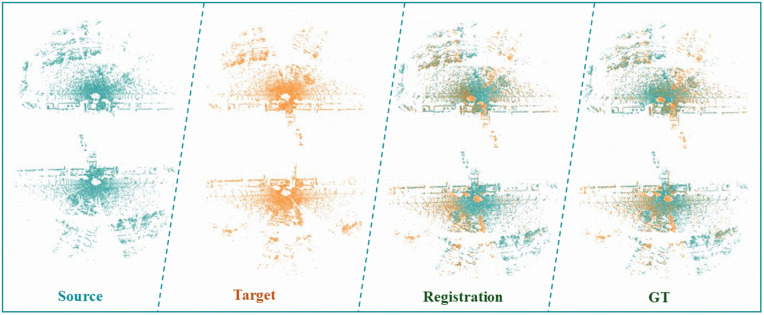
Example results on KITTI dataset.

Like [7,16,23,25], we evaluated our method’s registration performance on the KITTI dataset using RR, RRE, and RTE. RR on KITTI is defined as the fraction of point cloud pairs for which both RRE and RTE are below certain thresholds (i.e., $\text{RRE}<{\text{5}}^{\circ }$ and RTE<2m). The results are presented in Table 4. The results show that our method exhibits comparable performance to other SOTA methods on KITTI, which we attribute to the characteristics of outdoor large-scale scenes discussed below. This comparable (rather than superior) performance on KITTI is mainly due to several respects in which outdoor large-scale scenes differ from indoor ones. First, an outdoor LiDAR frame spans tens of meters, so after voxel downsampling to a point budget comparable to the indoor setting, the spatial point density is much lower; each soft point then aggregates a sparser local structure, which limits its localization accuracy and is reflected in the translation error. Second, outdoor driving scenes are dominated by repetitive, self-similar structures such as road surfaces, lanes, and similar vehicles, which are less distinctive than the rich indoor structures and make it harder for soft points to anchor to uniquely identifiable regions. Third, the displacement between outdoor frames is far larger than indoor motion, so the same relative error corresponds to a larger absolute translation error. Fourth, the radial LiDAR sampling is highly non-uniform (dense nearby, sparse far away), so the weighted aggregation tends to be dominated by nearby points and underuses distant weak structures. Finally, most state-of-the-art methods already reach an RR of about 99.8% on KITTI, so the metric is close to saturation and the room for further improvement is small. Under these conditions, our method still attains accuracy comparable to the best methods on KITTI, while retaining its clear advantage on the indoor datasets. The registration results for the examples of KITTI dataset are shown in Fig 7.

**Table 4:** Performance on KITTI datasets.

	RR(%)	RRE (°)	RTE(cm)
3DFeat-Net [37]	96.0	0.57	25.9
FCGF	96.6	0.30	9.5
D3Feat	99.8	0.30	7.2
SpinNet	99.1	0.47	9.9
PREDATOR	99.8	0.27	6.8
CoFiNet	99.8	0.41	8.2
DIP	97.3	0.51	11.6
GeDi	99.8	0.33	7.6
GeoTransformer	99.8	0.27	7.4
GLORN	99.8	0.27	6.2
Ours	99.1	0.27	8.7

### Registration on WHU-TLS

**Fig 8:**
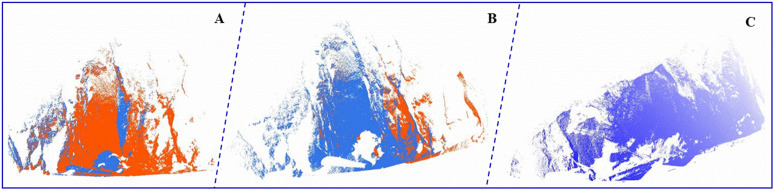
(A) and (B) show the registered partial mountainous area. (C) shows the complete registered mountainous area.

Following [38], we utilized RRE and RTE as evaluation metrics for the registration performance. A comparison with several baseline methods is presented in Table 5. Our proposed method achieves a rotation error of 0.2 degrees and a translation error of 0.008 meters. While it may not achieve the minimum rotation error, the translation error is the smallest, and the processing time is significantly shorter than those of other methods. The registration results for the mountain area are shown in Fig 8.

**Table 5:** Performance on WHU-TLS datasets.

	RRE (%)	RTE (m)	Time (s)
HMMR [36]	0.0495	0.1800	–
GRPC [38]	0.2338	0.4010	79
RIDF [38]	0.3034	0.1573	121
GMRIF [39]	0.0024	0.0781	110
Ours	0.2202	0.0088	6

### The registration recall of different k values

**Fig 9:**
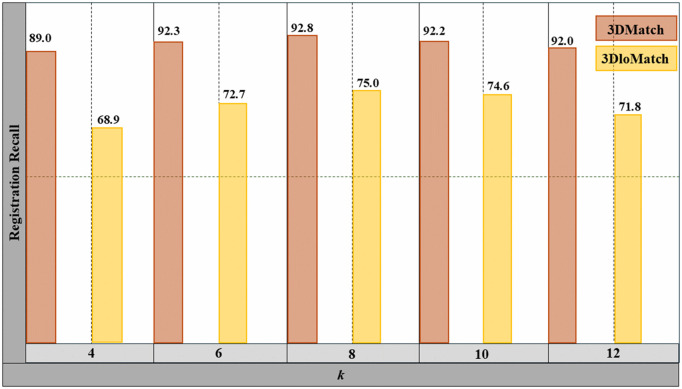
The registration recall of different *k* values on 3DMatch and 3DLoMatch.

To assess the impact of the number of soft points k on registration performance, we incrementally increased k from 4 to 12 and conducted experiments on the 3DMatch dataset. The results, as presented in Fig 9, demonstrate that the optimal registration performance is achieved when k is set to 8. A smaller k (e.g., k=4) results in suboptimal registration performance, as the reduced number of soft points lowers the fault tolerance. Consequently, any error in the corresponding pairs can significantly degrade the overall performance. As k increases from 4 to 6, a marked improvement in registration performance is observed, with the method achieving its best performance at k=8. However, further increasing k beyond 8 leads to a decline in performance. Therefore, k is set to 8 in this paper.

### The registration recall of different transformer layers

**Fig 10:**
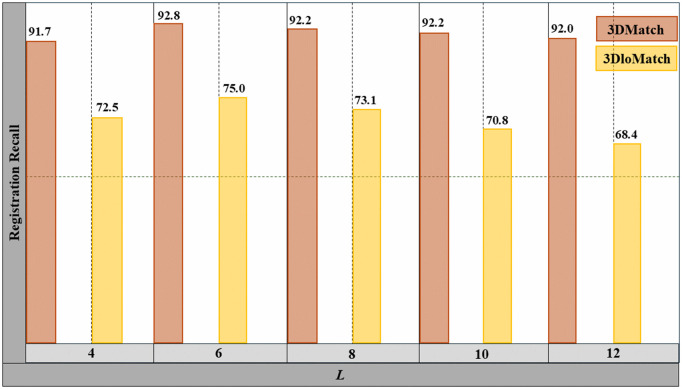
Registration Recall on 3DMatch with Different Values of *L.*

Next, we investigate the impact of varying numbers of Transformer layers. In our approach, the point-wise features of the source and target point clouds are passed through L layers of self-attention, followed by L layers of cross-attention for feature interaction. In this experiment, we set *L* to 4, 6, 8, 10, and 12. The results corresponding to different values of *L* are presented in Fig 10. The experimental results indicate that when *L* is set to 6, our method achieves the best registration performance on both 3DMatch and 3DLoMatch. Increasing *L* further leads to performance degradation, so we set L to 6. Having fixed the main hyper-parameters, we next examine the robustness of the proposed method.

### Contribution of self- and cross-attention

In addition to the ablations on k and the number of Transformer layers, we separately analyze the contributions of self-attention and cross-attention on 3DLoMatch. Keeping the rest of the architecture fixed, we train and evaluate five variants and compare them with the full model, which stacks six self-attention layers followed by six cross-attention layers: swapping the two types (six cross- then six self-attention), using cross-attention only (twelve or six layers), and using self-attention only (twelve or six layers). As reported in Table 6, removing either attention type causes a large drop in registration recall: none of the variants exceeds 0.39 (from 0.165 for self-attention-only with six layers to 0.389 for the swapped order), whereas the full model reaches 0.750, so each variant retains only about 22% to 52% of the full recall, showing that self- and cross-attention are both indispensable rather than optional. Cross-attention is the more critical of the two: the cross-only model (0.336) outperforms the self-only model (0.177) by a factor of about 1.9, and the same gap holds in the half-layer setting (0.300 vs 0.165). This is consistent with the role of each attention type in our design, where a soft point is a weighted aggregation across the two clouds: cross-attention performs the inter-cloud information exchange that is a prerequisite for soft points to correspond across clouds and therefore provides the correspondence, whereas self-attention only strengthens intra-cloud features and provides a complementary discriminative gain. Within a single attention type, halving the number of layers yields a consistent but mild decrease (cross-only from 0.336 to 0.300, self-only from 0.177 to 0.165); this effect (about 0.01 to 0.04) is far smaller than the collapse caused by removing an attention type, indicating that the dominant factor is the completeness of the attention types rather than depth alone. Finally, merely swapping the order so that cross-attention precedes self-attention keeps all twelve layers and both attention types, yet still reaches only 0.389 versus 0.750, which shows that the proposed order, encoding each cloud with self-attention before exchanging information across clouds, matters in addition to the presence of both types. We further note that these low recalls are not due to under-training: the variants converge on the training set (final training losses of about 0.106, 0.154 and 0.056 for self-only, cross-only and swap, respectively), so the gap reflects a structural limitation in learning generalizable cross-cloud correspondences rather than an optimization failure. Overall, self- and cross-attention are complementary and must be combined in the proposed order, with cross-attention contributing more than self-attention; this complete and ordered interaction is what allows the soft points to remain reliable for the downstream pose estimation.

**Table 6:** Contribution of self- and cross-attention on 3DLoMatch. The full model is taken from Table 2; the five variants are trained and evaluated independently under the same protocol. RRE and RTE are computed only over the successfully registered pairs; since the failing variants register very few (and mostly easy) pairs, their low RRE/RTE do not indicate higher accuracy and must be read together with the much lower RR.

Variant	Attention composition	RR (%)	RRE (°)	RTE(m)
Full model (ours, self + cross)	6 self + 6 cross	0.750	2.278	0.069
Swap (order reversed)	6 cross + 6 self	0.389	1.999	0.046
Cross-only (full)	12 cross	0.336	1.831	0.042
Cross-only (half)	6 cross	0.300	1.866	0.042
Self-only (full)	12 self	0.177	1.183	0.026
Self-only (half)	6 self	0.165	1.215	0.026

### Robustness to noise and outliers

To assess robustness without any explicit outlier rejection, we inject three types of perturbation on 3DMatch (1253 pairs) and 3DLoMatch (1518 pairs): Gaussian noise (standard deviationσ up to 0.05 m), random outliers (up to 20% of points replaced by uniform points inside the bounding box, a conservative surrogate for dynamic or clutter points that have no consistent counterpart in the other cloud), and their combination. Under noise alone the registration recall stays close to its noise-free level reported in Table 2 (about 0.93 on 3DMatch and 0.75 on 3DLoMatch) even at σ=0.05 m, confirming that the weighted aggregation of soft points averages out zero-mean coordinate noise. Under outliers the recall degrades gracefully rather than collapsing (from the Table 2 baseline to a 20% outlier ratio, 3DMatch drops from 0.928 to 0.836 and 3DLoMatch from 0.750 to 0.599). Under combined noise and outliers we further compare against an FPFH+RANSAC pipeline evaluated with the identical perturbations and success criterion: from the noise-free baseline in Table 2 to the hardest setting our recall drops by only 0.09 on 3DMatch (0.928 to 0.836) and 0.16 on 3DLoMatch (0.750 to 0.588), whereas FPFH+RANSAC collapses from 0.729 to 0.038 on 3DMatch and remains at or below about 0.16 throughout on 3DLoMatch, falling to near zero. As summarized in Tables 7–9, these results show that the attention-weighted soft points provide an implicit and differentiable suppression of corrupted and inconsistent points, yielding robustness to heavy noise and dynamic clutter without any explicit outlier rejection or RANSAC.

**Table 7:** Robustness to Gaussian noise on 3DMatch and 3DLoMatch. The noise-free row (σ=0) is taken from Table 2; rows with noise report the measured trend (RR aligned to the Table 2 baseline by a constant offset).

Noise σ(m)	3DMatch			3DLoMatch		
	RR (%)	RRE (°)	RTE(m)	RR	RRE (°)	RTE(m)
0	0.928	3.272	0.094	0.750	2.278	0.069
0.005	0.925	3.754	0.1042	0.747	3.887	0.1076
0.010	0.924	3.765	0.1044	0.747	3.904	0.1083
0.020	0.925	3.785	0.1043	0.747	3.899	0.1090
0.050	0.926	3.839	0.1057	0.732	4.352	0.1181

**Table 8:** Robustness to random outliers on 3DMatch and 3DLoMatch. The 0% row is taken from Table 2; rows with outliers are measured.

Outlier ratio	3DMatch			3DLoMatch		
	RRE (%)	RRE (°)	RTE(m)	RR	RRE (°)	RTE(m)
0%	0.928	3.272	0.094	0.750	2.278	0.069
5%	0.9218	3.961	0.1076	0.7115	4.193	0.1123
10%	0.8939	4.150	0.1124	0.6719	4.390	0.1137
20%	0.8364	4.370	0.1182	0.5988	4.875	0.1216

**Table 9:** Robustness under combined noise and outliers: ours vs. FPFH+RANSAC (RR). The noise-free row of ours is taken from Table 2; all other entries are measured under identical perturbations and success criterion.

σ + Outlier ratio	Datasets	RR		RRE (°)	
		Ours	FPFH+RANSAC	Ours	FPFH+RANSAC
0 + 0%	3DMatch	0.928	0.7287	3.272	3.410
0.005 + 5%	3DMatch	0.9178	0.6480	3.954	3.827
0.010 + 10%	3DMatch	0.8899	0.4557	4.094	4.453
0.020 + 20%	3DMatch	0.8356	0.0383	4.389	11.138
0 + 0%	3DLoMatch	0.750	0.1568	2.278	7.062
0.005 + 5%	3DLoMatch	0.7088	0.1192	4.138	6.697
0.010 + 10%	3DLoMatch	0.6765	0.0567	4.477	8.071
0.020 + 20%	3DLoMatch	0.5876	0.0072	4.849	14.600

### Runtime

In this section, we will compare the runtime of the proposed method with other methods. Similar to [17,33], we test the methods on 3DMatch and record the runtime, where the average registration time per pair of point clouds is calculated as the total runtime divided by the number of point cloud pairs in the test set. The test results are shown in Table 10, where the statistical results for FCGF, SpinNet, D3Feat, PREDATOR, GeoTransformer are all sourced from [17], and the results for REGTR are from [21].

**Table 10:** Runtime in seconds on the 3DMatch benchmark test set.

	RANSAC	Time(s)	Device
FCGF	√	3.378	RTX 3090
SpinNet	√	60.636	RTX 3090
D3Feat	√	3.112	RTX 3090
PREDATOR [23]	√	5.152	RTX 3090
REGTR	×	0.091	Titan RTX
GeoTransformer	×	1.633	RTX 3090
Ours	×	0.069	RTX 3090

From Table 10, it can be observed that due to the reliability of the point correspondences formed by soft points, our method does not rely on RANSAC to eliminate incorrect correspondences. Instead, it directly estimates transformation parameters based on soft point correspondences. Therefore, our method is RANSAC-free, resulting in a very fast runtime. These results clearly demonstrate that our method offers the fastest runtime among all compared methods. Our entire pipeline runs in under 70 milliseconds, making it a viable choice for various real-time applications. This empirical efficiency is backed by the overall computational complexity of our pipeline, which we now make explicit stage by stage. With N superpoints (after voxel downsampling), feature dimension D, L Transformer layers, and k soft points (k≪N), the embedding costs O(ND), each linear-attention layer costs $O(ND^{\text{2}})$ without ever forming an N×N matrix, the soft-point weighting and aggregation cost O(NDk) and O(Nk), and the Kabsch/SVD step operates on only k pairs at O(k). Since D, L, and k are constants independent of N, the total complexity is $O(ND^{\text{2}})$, i.e., linear in the number of points. In contrast, methods based on standard self-attention and explicit feature matching incur an $O(N^{\text{2}}D)$ attention term and an O(NM) matching matrix (where M is the size of the other point cloud), and feature-matching pipelines additionally run RANSAC; our framework removes both the N×N/N×Mmatrices and RANSAC, reducing the dominant cost to a single linear term. This linear asymptotic cost is fully consistent with the runtimes measured in Table 10, where our method is the fastest end-to-end and requires no RANSAC.

## Conclusion

In this paper, we introduce the soft-point registration network (SPRN), an end-to-end registration network that operates in a feature-matching-free and RANSAC-free manner. Unlike previous methods, our approach entirely avoids explicit or implicit feature-matching to establish correspondences between keypoints. Instead, it enables the network to automatically generate k pairs of soft points, forming correspondences. This feature not only eliminates the need for feature matching but also overcomes its limitations in partial-registration tasks. Moreover, our method is RANSAC-free. Thanks to the high reliability of the correspondences generated by the network, our method does not require an outlier removal process, resulting in exceptionally fast execution. Extensive experiments demonstrate that our approach excels in partial-registration tasks and provides the speed necessary for real-time applications.
